# Single-Amplicon Sequencing of Coding-Complete Enteroviruses and Rhinoviruses Genomes from Clinical Specimens

**DOI:** 10.64898/2026.10.19.760066

**Published:** 2026-10-21

**Authors:** Stephanie Goya, Preston C Longley, Clarice Mauer, Alexander Greninger

## Abstract

Rhinoviruses and enteroviruses are among the most frequently detected respiratory viruses, yet routine diagnostics do not report species or genotype, and standard VP1 genotyping cannot resolve transmission chains or recombination. Recovering sequence beyond VP1 currently requires probe-based enrichment, deep metagenomic sequencing, or several overlapping amplicons. We developed a single-amplicon PCR and sequencing workflow that generates coding-complete genomes from both enteroviruses and rhinoviruses in a single reaction. We evaluated the protocol on 140 rhinovirus-positive respiratory specimens and a panel of 41 isolates spanning 26 enterovirus A-D types, comparing it with hybridization capture sequencing and relating its performance to viral load measured by RT-qPCR and droplet digital RT-PCR. Single-amplicon sequencing recovered 116 high-quality rhinovirus genomes, across 57 rhinovirus genotypes, compared with 113 from hybridization capture. Single-amplicon sequencing was associated with higher fidelity and more uniform sequencing depth compared to hybridization capture. The same primer configuration also recovered two unexpected coxsackieviruses (A6 and A16) from rhinovirus-positive clinical specimens, and the expected polyprotein-coding sequences from all 40 enterovirus isolates tested. Recovery increased with viral abundance, and a visible amplicon band predicted sequencing success. Single-amplicon sequencing reagents cost approximately three-fold less per sample than hybridization capture ($30 versus $85) and required one-third as much time. These findings support single-amplicon sequencing as a simple, cost-effective approach for genomic surveillance of enteroviruses and rhinoviruses from clinical specimens.

**Importance:**

Routine respiratory virus tests can detect rhinoviruses and enteroviruses but seldom identify the circulating viral lineage, while VP1 genotyping of rhinoviruses and enteroviruses cannot adequately resolve transmission or recombination. Genome sequencing can provide this information, but existing approaches may require extensive sequencing, probe-based enrichment, or multiple overlapping PCR products. We describe a single-round, single-amplicon method that recovers the polyprotein-coding region across both rhinoviruses and enteroviruses. The method produced highly uniform coverage and reproducible consensus sequences at approximately one-third the price of hybridization capture, and it includes a simple pre-sequencing quality check. This streamlined workflow can help make enterovirus and rhinovirus genomic surveillance more accessible to clinical and public health laboratories.

